# Abstracts from Foreign Journals

**Published:** 1900-06

**Authors:** 


### ABSTRACTS FROM FOREIGN JOURNALS.

PASSAGE OF THE BACILLUS OF KOCH INTO THE MILK OF A TUBERCULOUS WOMAN. By Drs. H. Roger and M. Garnier. Translated from the *Comptes Rendus de la Société de Biologie*, vol. lii., No. 8, March 2, 1900. (From the Laboratory of the State Live-stock Sanitary Board of Pennsylvania.) The transmission of tuberculosis by milk has been studied especially in the bovine species, the importance of cow's milk as a food product leading authors to investigate whether or not this secretion could contain the bacillus of Koch. It was soon recognized that the passage of the bacillus into the milk took place quite frequently, even in the absence of mammary tubercles. If a cow is at all tuberculous her milk should be considered suspicious.

It is generally admitted that these results do not apply to the human race, and that the milk of tuberculous women never contains the bacilli.

Escherich, in researches now old, examined the milk of women with tuberculosis of the lungs without ever finding the microörganisms, but it seems that he made no inoculations. Fede, in 1892, studied the question in a more thorough manner. He injected the milk of tuberculous women into rabbits and guinea-pigs subcutaneously, intraperitoneally, or into the anterior chamber of the eye; never did he observe the development of tubercles. These results have been confirmed by Bonis. Bang has never obtained positive results by inoculation.

We have observed a fact which contradicts the classic opinion and proves that the milk of phthisical women may sometimes be virulent. A woman, aged thirty-four years, entered the hospital February 16, 1899, on account of a tuberculous pharyngitis. Her general condition at this time was fairly satisfactory, but besides the pharyngeal lesion albuminuria was discovered, and auscultation revealed induration at the apex of the left lung. The patient was pregnant and near her term; delivery took place under good conditions on March 7th following. From this moment the symptoms grew worse, the pulmonary lesions increased rapidly, and ten days after delivery the existence of a cavity at the summit of the left lung was determined. Then the general condition grew more and more bad, the fever was higher, and the woman died on March 24th, seventeen days after her confinement. At the autopsy advanced tuberculous lesions were found in the summits of both lungs ; the apex of the left contained a large cavity ; the entire pulmonary parenchyma was studded with gray granulations, as were also the liver, the kidneys, and the thyroid gland.

The baby born at term weighed 2685 grammes. Two days after birth it showed jaundice, then a green diarrhœa set in, followed by an œdema, hard, most marked in the feet and hands, which soon extended up the legs and thighs. The baby had from the outset been fed from the bottle, but seeing it about to perish the mother wished to nurse it, so from March 10th to 12th she gave it the breast, but the aggravation of her condition did not permit of its continuance. Fed on the bottle the child survived six weeks. Its weight, taken regularly every three days, underwent remarkable oscillations ; it reached 2950 grammes on April 9th, but soon fell to 2620, again reached 2900, and finally fell to 2330 on April 25th, the eve of its death. At the autopsy numerous granulations were found in the liver, the spleen, the kidneys, the mesenteric glands.

Microscopical examination of sections of the liver showed typical tuberculous granulations in which the bacillus of Koch could be stained.

On March 11th, four days after confinement, two guinea-pigs had been inoculated with milk drawn aseptically from the mother's breast. The first pig, weighing 440 grammes, received 4 cubic centimetres of milk under the skin ; only 2 cubic centimetres were injected into the peritoneal cavity of the second, which weighed 525 grammes. The first died very much emaciated on April 14th ; it showed typical lesions of generalized tuberculosis ; the liver and spleen were filled with tubercles ; the lungs were studded with gray granulations. The other pig survived. On April 21st it weighed 565 grammes ; June 14th its weight reached 650 grammes, but the glands of the groin were found to be hypertrophied. It was killed on January 28th last, weighing then 630 grammes, and its condition was excellent. At the autopsy a fibrous peritonitis was found corresponding to the point of inoculation ; the coils of intestines were adherent to each other and to the lateral wall of the abdomen ; the posterior wall was free ; the omentum was large and retracted and without visible tubercles. The rest of the abdomen did not show any lesions. The surface of the liver was dotted with depressed points resembling cicatrices ; the spleen appeared healthy ; the suprarenal capsules were enlarged. Microscopical examination of the liver, the spleen, the thyroid gland and an hypertrophied gland found in the groin of the side opposite to the peritonitis showed neither tuberculous granulations nor sclerous changes; finally, the search for the bacillus both in sections and in the juice of the gland taken at the autopsy was negative.

Of our two guinea-pigs only one then became tuberculous, the one which had received the larger dose of milk; in this animal tuberculosis developed according to its usual method, and caused death in thirty-three days.

The other, which had received only one-half the quantity, survived, and appeared to be in excellent condition when killed, more than ten months after the inoculation. However, it was not entirely uninjured; the milk injected had not proved to be an entirely innocuous fluid, and the fibrous peritonitis which had developed at the point of inoculation gave evidence of a pathological process. This was not confined entirely to the peritoneum; an hypertrophied gland in the groin and cicatrices on the surface of the liver showed that there had been an attempt at generalization. The history of the first pig enabled us to explain the lesions found in the second. In the first case the quantity of bacilli injected was great enough to overcome the resistance of the animal, and the tuberculosis became generalized. In the second, on the contrary, the dose injected having been less, the animal overcame the infection. It recovered from the lesions produced, and we found only harmless cicatrices without any characteristic features.

The milk which we injected therefore contained bacilli, and was capable of transmitting tuberculosis. The child, having taken the milk of the mother for two days, could have been infected by this alone. Let us note that it died only twelve days after the first pig, which was injected four days after the birth of the infant. It is certain that besides the nursing the child was exposed to other sources of infection, for leaving out of consideration a possible intra-uterine contagion we must remember that the mother had a pharyngeal ulceration, and that bacilli were abundant in her mouth. However, since the autopsy showed that the lesions predominated in the mesenteric glands, the liver and the spleen, it is probable that the digestive tube was the principal if not the only port of entry.

The milk of a tuberculous woman can, therefore, serve as a vehicle for the bacillus of Koch, even in the absence of any tuberculous lesions of the mammary glands that can be detected clinically.

RABIFORM SYMPTOMS DUE TO SPIROPTERA. Translated from *Recueil de Méd. Vét.*, March 30, 1900, vol. vii., No. 6, Series viii. At a meeting of La Société Centrale de Médecine Vétérinaire, on March 8, 1900, Monod, military veterinarian at Hanoi, Tonkin, presented notes of an interesting case. On January 3, 1900, the cadaver of a dog was brought to him for examination. The animal was of notably good disposition, but after several days of dulness had, in December, bitten his owner, then a boy, who attempted to tie him up, and another dog in the same house; and finally he attacked a person who was fortunately protected from injury by his clothing. Soon after tremors of the hind-quarters were noticed, followed by complete paralysis. He had refused all nourishment for twenty-four hours, and was found dead in the morning where he was confined.

At the autopsy the mouth was found filled with bloody saliva, the tongue was cut by the teeth, the stomach empty, but acutely congested and showing punctate hemorrhages. Similar lesions were found along the intestine, and also, floating in a blackish and disgusting liquid, were numerous spiroptera, hairs swallowed by the animal in licking itself, and bits of wood. In the œsophagus, 5 or 6 centimetres from the stomach, a small opening was seen, from which emerged half of a spiroptera, the other part remaining in a canal which communicated with a voluminous tumor containing numbers of the parasites of all sizes.

Several rabbits were inoculated with the medulla of the dog, and have remained well up to the 26th, from which it is concluded that the dog did not suffer from rabies in spite of his alarming symptoms, which must be attributed entirely to the tumor of spiroptera.

In the discussion, Raillet said that as early as 1830 Warren had noted the presence of the spiroptera sanguinolenta in dogs supposed to have died of rabies in the Island of Malta. In 1853 Rangé wrote: "As the result of many observations, it appears that spiroptera in the œsophagus of dogs may cause symptoms entirely analogous to those of rabies." This observation may be explained by the fact that in making post-mortems on dogs supposed to be rabid it is the rule to examine the digestive tract very carefully, and it has not been shown heretofore that the symptoms observed were not due to rabies. Dr. Monod deserves credit for having established the fact that the presence of spiroptera in the œsophagus can cause symptoms like those of rabies, but which have no real connection with the true disease.

The spiroptera sanguinolenta is a nematode worm, readily recognized by its blood-red color. The male is from one and a quarter to two inches in length, has a spiral tail furnished with two lateral wings, each of which is supported by six papillæ. The female is two and one-quarter to three inches in length, and the tail is only slightly curved. The spiroptera is found almost exclusively in tumors in the wall of the stomach and œsophagus, which vary in size from a hazel-nut to a pigeon's egg, and are dense to the feel. Only from one to three tumors are usually found, and each tumor contains from two to twenty worms, coiled and embedded in a purulent fluid, which may be made to exude by pressure from the orifice by which the tumor communicates with the digestive tract. It is hoped that in order to clear up this point this observation will lead veterinarians in this country to make careful examinations in cases of dogs dying with symptoms of rabies.

---

"The Value of Glycerinized Lymph over Vaccine Points, with a Series of Collective Reports," is the title of an article prepared by Dr. Albert C. Barnes, of Philadelphia. In summing up the relative value of these forms he states: 1. Properly prepared glycerinized vaccine is pure and free from staphylococci, streptococci, and other pathogenic organisms which are invariably found (Copeman, Crookshank, Pfeiffer, Reed, U. S. A.) on vaccine points. 2. Glycerinized vaccine affords absolute protection against smallpox; vaccine points are uncertain in this regard. 3. Vaccination with glycerinized products does not cause excessive inflammation of the vaccinated area. Cellulitis and inflammation of the lymph vessels and glands, amounting at times to abscess formation, are not infrequent sequences of the use of vaccine points. 4. Vaccine points are apt to lead to a false sense of security, inasmuch as they induce a local staphylococcic or streptococcic infection which is entirely distinct from true vaccination. Such a result is not protective against smallpox. 5. A high estimate of successful take from vaccine points is by these and numerous other reports shown to be not over 60 per cent. in primary cases and a much lower percentage in secondary cases. 6. Glycerinized vaccine has been officially adopted by the governments and health authorities of the United States, Great Britain, Germany, France, Russia and Belgium. It should be universally adopted in private practice.

